# Multidisciplinary management of ovarian germ cell tumours—a single institutional study from India

**DOI:** 10.3332/ecancer.2021.1290

**Published:** 2021-09-14

**Authors:** Sandip Ganguly, Sumedha Gargy, Archisman Basu, Meheli Chatterjee, Anik Ghosh, Basumita Chakraborti, Bivas Biswas, Deepak Dabkara, Shweta Rai, Arunava Roy, Sonia Mathai, Jaydip Bhaumik, Joydeep Ghosh

**Affiliations:** 1 Department of Medical Oncology, Tata Medical Center, 14 MAR (E-W), New Town, Rajarhat, Kolkata, West Bengal 700156, India; 2 Department of Gynaecology, Rajendra Institute of Medical Sciences, Ranchi 834009, India; 3 Department of Gynaecologic Oncosurgery, Tata Medical Center, 14 MAR (E-W), New Town, Rajarhat, Kolkata, West Bengal 700156, India

**Keywords:** ovary, germ cell, surgery, chemotherapy

## Abstract

**Background:**

Ovarian germ cell tumours constitute a heterogeneous group of neoplasm with malignant potential being seen in 5% of cases. There is limited data on treatment outcomes of patients with malignant ovarian germ cell tumours (MOGCT). Here, we present our hospital audit of patients with MOGCT.

**Material and methods:**

This is a retrospective data review of patients with MOGCT treated between May 2011 and December 2019. Patients were treated with staging laparotomy and adjuvant chemotherapy, wherever applicable. Surveillance was allowed for those at low risk for recurrence. Clinicopathologic features and treatment details were recorded, and survival analysis was performed.

**Results:**

Sixty-five patients with a median age of 25 years (range: 11–52 years) were treated during the study period. The most common histology was immature teratoma in 35.3% of cases. International Federation of Gynecology and Obstetrics stage IC was the most common stage of presentation (47%). Surveillance was advised for 12.3% of cases. Systemic therapy was given in 51 (78%) patients. At a median follow-up of 46 months (range: 1–109 months), the median progression-free survival (PFS) was not reached. Five-year PFS was 79.3% (95% CI: 65.8–88). The most common toxicity was febrile neutropenia (22%) among those who received systemic therapy.

**Conclusion:**

Immature teratoma was the most common histology in our series. The majority presented in the early stage. MOGCT is a highly curable disease with surgery and systemic therapy.

## Introduction

Malignant ovarian germ cell tumours (MOGCT) comprise less than 5% of all ovarian malignancies in the western countries. In contrast to epithelial ovarian cancers, they commonly occur in the second and third decades of life [1]. Advancement in systemic therapy and surgical techniques has led to a marked improvement in survival of patients with MOGCT. The fertility preservation approach at the time of surgery and avoidance of toxicity with systemic chemotherapy play a crucial role in the treatment of MOGCT [2]. Because of the rarity of these tumours, there is limited published literature regarding the clinical profile and outcome of MOGCT. Here, we report the clinicopathologic profile, treatment and outcome along with the treatment-related toxicity in patients with MOGCT from our institute.

## Materials and methods

### Patients

This is a single institutional collaborative retrospective study involving two departments, Department of Medical Oncology and Department of Gynaecologic Oncology of the hospital. Physicians and surgeons in both the departments were actively involved in concept design, data acquisition, manuscript writing and editing. Patients with MOGCT registered and treated in the Department of Medical Oncology and Gynaecological Oncology of our institute from May 2011 to December 2019 were included in the study. Patients with mature teratoma and secondary somatic transformation of malignant teratoma to other histology were excluded from the study. Those who received at least one cycle of systemic chemotherapy or those who were kept under surveillance and had more than one outpatient follow-up visit with serum tumour markers were included for the survival analysis. Clinicopathologic features and treatment details were retrieved from electronic medical records and analysed for all patients. Being a retrospective audit, consent waiver was obtained from the Institutional Review Board (EC/WV/TMC/38/20) as per the institutional policy.

### Investigations

All patients underwent pre-operative abdominal imaging and serum tumour markers – Alpha-fetoprotein (AFP), beta-human chorionic gonadotropin (b-HCG) and lactate dehydrogenase (LDH). For patients with normal tumour markers with an ovarian mass in abdominal imaging, the diagnosis of MOGCT was made by the histopathological examination of the operated specimen. Staging workup was done with contrast enhanced computed tomography of thorax and abdomen.

### Treatment

After confirmation of diagnosis and staging workup, the patients were discussed in the multidisciplinary tumour board meetings for further management. For those with resectable early stage disease, surgery was the primary treatment. Initial surgical intervention varied widely as many patients get referred to our centre after initial management from outside centres. Some patients had complete staging surgery, but some underwent cystectomy or piecemeal excision of the ovarian tumour and were referred to our centre for further management. Completion staging surgery was offered to patients who have undergone incomplete surgery before coming to our hospital. Patients with operable stage I dysgerminoma and grade I immature teratoma were observed while adjuvant chemotherapy was given to other patients. Primary chemotherapy followed by complete surgery was the preferred treatment among those with unresectable disease at initial presentation. Chemotherapeutic regimens were EP (intravenous etoposide 100 mg/m^2^ on days 1–5, cisplatin 20 mg/m^2^ on days 1–5 every 21 days) or BEP (intravenous etoposide 100 mg/m^2^ on days 1–5, cisplatin 20 mg/m^2^ on days 1–5, intravenous bleomycin 30 U iv push on days 1, 8 and 15 every 21 days). Salpingo-oophorectomy (SO) with omentectomy and nodal sampling and excision of any residual deposits were the main components of interval debulking surgery. Hysterectomy was done in patients who did not want to preserve fertility. Patients were followed up with tumour markers for response assessment. Patients with persistently raised tumour markers and gross residual progressive diseases were treated either with salvage surgery in combination with salvage chemotherapy or salvage chemotherapy alone in cases of the inoperable disease. Salvage surgery involved removal of contralateral ovary and uterus along with omentectomy, lymphadenectomy and excision of visible tumour deposits. In second-line or salvage settings, chemotherapeutic protocols used were TIP (intravenous paclitaxel 250 mg/m^2^ continuous infusion over 24 hours on day 1, ifosfamide 1.5 gm/m^2^ on days 2–5, intravenous cisplatin 25 mg/m^2^ on days 2–5 every 21 days) or VeIP (intravenous etoposide 100 mg/m^2^ on days 1–5, ifosfamide 1.2 gm/m^2^ on days 1–5, intravenous cisplatin 20 mg/m^2^ on days 1–5 every 21 days) with myeloid growth factor support. Toxicity to chemotherapy was measured by Common terminology criteria for adverse events version 4.03 [3].

The patients were followed up for acute and long-term treatment related toxicities as well as for recurrence. Those who had weight gain on follow-up were assessed for symptoms and signs of metabolic syndrome including laboratory parameters as per American Heart Association/National Heart, Lung, and Blood Institute Scientific Statement [4]. Those who had full component or partial component of metabolic syndrome were subjected to appropriate intervention [5] – weight reduction, regular exercise, life style modification and use of statin whenever indicated and they were further followed up for response to intervention.

### Statistical analysis

Descriptive statistics were used for clinicopathologic characteristics. Survival was estimated with Kaplan–Meier (KM) method and data were censored on 1 June 2020. Progression-free survival (PFS) was calculated from the date of diagnosis to the date of clinical and/or radiological disease progression. Overall survival (OS) was calculated from the date of diagnosis to the date of death from any cause. Patients who were lost to follow-up were censored at the date of last contact/follow-up. Patients who were alive on 1 June 2020 were censored for OS analysis.

Patients who were lost to follow-up or who had abandoned treatment were also included in the event-free survival and OS analyses, and the outcomes for these patients were confirmed by telephone contact. Treatment abandonment was included in the survival analysis in the present study as it has been proposed that patients who do not comply with or who abandon treatment be included in survival analysis for studies from developing nations to provide a true picture of outcomes from these countries [6]. STATA/SE 11.0 (Stata Corp, College Station, Texas, USA) was used for statistical analysis.

## Results

A total of 65 patients have been registered in our department during the study period. The baseline characteristics of the patients have been shown in Table 1. The median age of the study population was 25 (range: 11–58) years. Only two patients presented with recurrent disease. Incidental diagnosis of MOGCT at the time of pregnancy was made in three (4.4%) patients. The most common histology was immature teratoma in 23 (35.3%) followed by yolk sac tumour in 22 (33.8%) patients. Thirty-two (47%) patients were diagnosed with MOGCT at different centres and did not have pre-operative serum tumour markers. The most common stage of the presentation was the International Federation of Gynecology and Obstetrics (FIGO) IC in 32 (47%) patients. The type of surgical modalities has been shown in Table 2. Upfront surgery was done on 52 (76.4%) patients. Unilateral SO (USO) was done in 25 (36.7%) patients. Nodal sampling done in 12 patients was found to be disease free on histopathological examination. Post-surgical morbidities were few.

Fifty-nine (87%) patients received either systemic chemotherapy or were kept under observation after surgery. The rest nine patients (13%) did not turn up for the same and these patients were excluded from the survival analysis. EP was the most common chemotherapeutic regimen used in 37 (63%) patients. The median number of cycles used was three (Table 1). Eight (14%) patients were kept under surveillance with serial serum tumour marker assessment. Patients kept on surveillance were serially monitored with tumour markers at intervals of 3 months for the first 2 years and then every 6 months.

Five patients had persistent elevation of serum AFP after completion of treatment. They were kept on follow-up as imaging did not reveal any disease and AFP normalised during subsequent follow-up in three patients. The other two patients received salvage chemotherapy. Of all the patients, salvage chemotherapy was given in five (8.4%) patients. Two patients had upfront immature teratoma and yolk sac tumour while one had mixed germ cell tumour (MGCT) who went on to receive salvage therapy.

**Figure 1.**
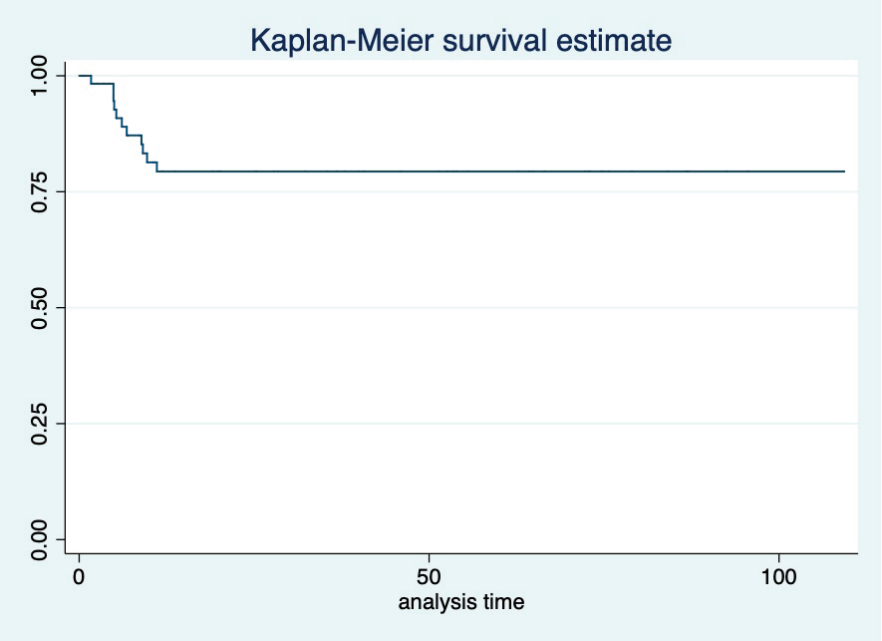
KM graph showing median PFS of the entire cohort.

**Figure 2.**
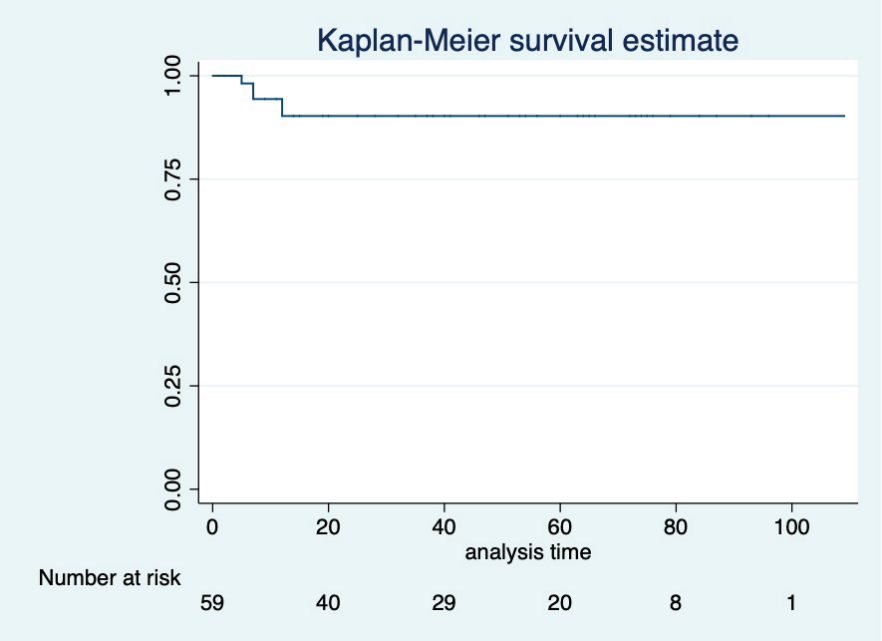
KM graph showing median OS of the entire cohort.

**Figure 3.**
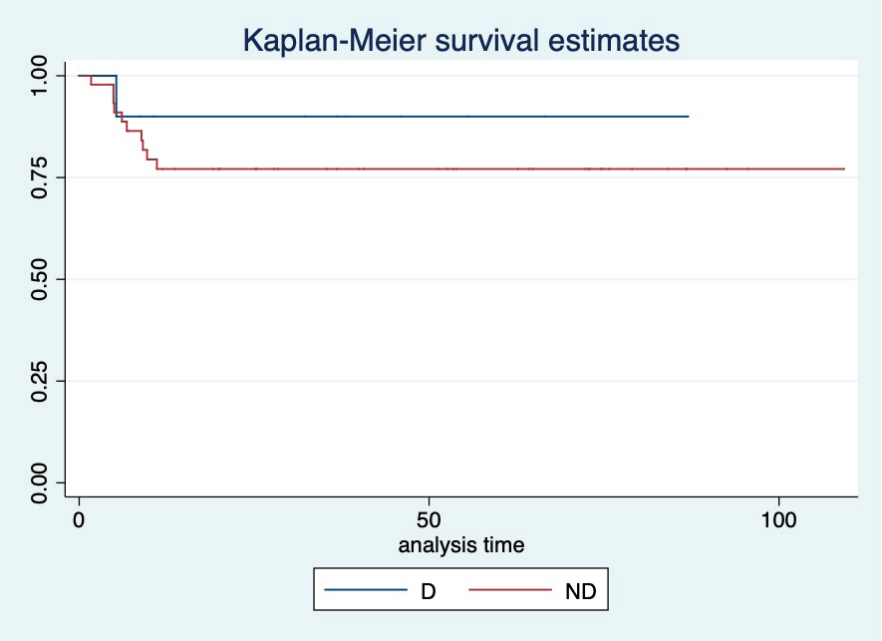
KM graph showing median PFS of patients with dysgerminoma (D) histology and non-dysgerminoma (ND) histology.

**Figure 4.**
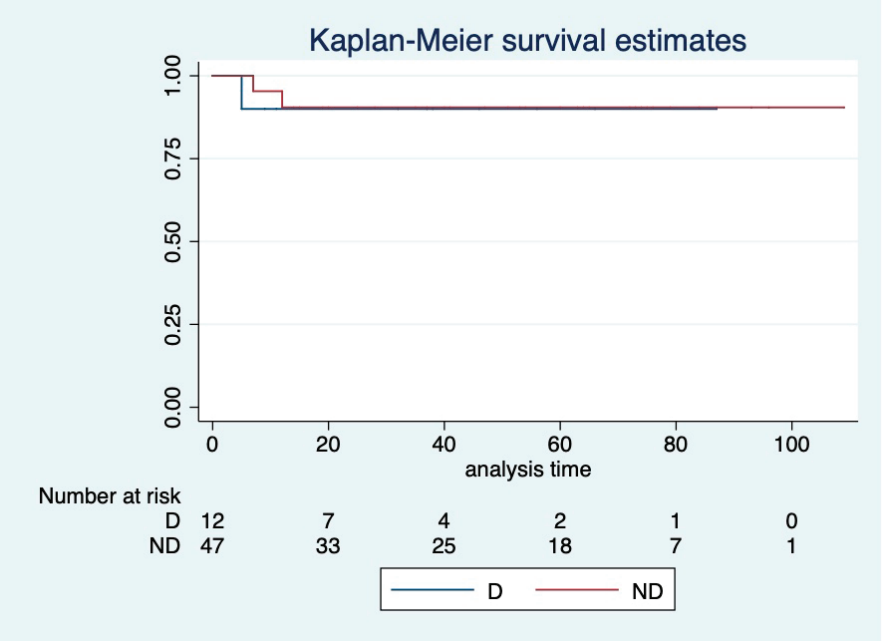
KM graph showing median OS of patients with dysgerminoma (D) histology and non-dysgerminoma (ND) histology.

At a median follow-up of 46 (95% CI: 32–60) months, the median PFS and OS of the overall study population were not reached. Five-year projected PFS was 79.3% (95% CI: 65.8–88) and the OS was 90.1% (95% CI: 77.8–95.8) as shown in Figures 1 and 2, respectively. Projected 5-year PFS for patients with dysgerminoma was 90% (95% CI: 47.3–98.5) and 77.1% (95% CI: 61.6–87.2) for non-dysgerminoma histology as shown in Figure 3. Projected 5-year PFS for immature teratoma grade 1 was 100%, immature teratoma grade 2 was 75% (95% CI: 12.7–96.3), immature teratoma grade 3 was 53.8% (95% CI: 25–76), yolk sac was 91% (95% CI: 68.3–95.8) and mixed germ cell tumour was 66.7% (95%CI: 54.1–95). Projected 5-year PFS for stage I, II, III and IV cancers was 91%, 100%, 53.4% and 66%, respectively. Projected 5-year OS for patients with dysgerminoma was 90% (95% CI: 47.3–98.5) and 90.2% (95% CI: 76.1–96.2) for non-dysgerminoma histology as shown in Figure 4. Projected 5-year OS for immature teratoma grade 1 was 100%, immature teratoma grade 2 was 100%, immature teratoma grade 3 was 92.3% (95% CI: 57–99), yolk sac was 86% (95% CI: 62.3–95) and mixed germ cell tumour was 100%. Projected 5-year OS for stage I, II, III and IV cancers was 100%, 100%, 70% and 66%, respectively.

The most common grade 3–4 toxicities were febrile neutropenia in thirteen (22.0%), peripheral neuropathy in seven (11.8%) and vomiting in three (5%) patients. One patient had grade 5 bleomycin induced pulmonary toxicity. Three (5%) patients developed features of metabolic syndrome.

## Discussion

Ovarian germ cell tumours comprise a heterogeneous group of diseases with malignant potential is seen in 3% of cases [1]. Though the incidence of MOGCT is less in the western countries, it is around 15% among Asians [7].

MOGCT is a malignant neoplasm of adolescents and young adults. The median age of the study population is around 20 years in most studies which is less compared to that of our study cohort [8, 9]. This can be explained by the fact that the paediatric patients in our hospital are treated by a separate paediatric oncology unit and hence, not included in our cohort. Abdominal pain was the most common presenting symptom among our patients which was similar to those in published literature [8, 10]. The most common histology in the study population was immature teratoma comprising 35.3% of total cases. This finding does not agree with the available literature where dysgerminoma has been the most common histology being reported in one study [11] while another one has reported mixed germ cell tumour as the most common histology [8]. Forty-seven percent of patients were diagnosed to have FIGO stage IC and this was similar to study by Agarwal *et al* [8]. But in the study done by Topuz *et al* [1], majority were in stage IA and in contrast to it most patients presented in stage III by Lakshmanan *et al* [11]. This variation in stage is mainly based on referral bias.

Baseline tumour markers were not available in a large proportion of our patients. This is because of the fact that many patients at first presented themselves to a general surgeon or a gynaecologist. They undertook surgical procedures on the adnexal mass without proper preoperative evaluation and only after the availability of the histopathology report, the patients got referred to a tertiary care cancer hospital. Among patients whose tumour markers were available, the mean AFP level in our group was 6,553.9 +/− 1,655 ng/mL. This was much higher compared to other studies where it was reported to be around 892.3 ng/mL [11]. This difference is explained by the fact that yolk sac tumour was the most common histology in our study.

Fertility preserving surgical (FPS) approach is recommended in patients with MOGCT [12]. Fifty-four (79.4%) patients underwent FPS which was more than that reported by Lakshmanan *et al* [11] where it was only 36.8% [11]. This difference can be explained by the fact that many of our patients presented in the early stage compared to their study where the advanced stage of the presentation was most common. Only 24 (35.2%) patients underwent omentectomy with or without lymphadenectomy. The role of lymphadenectomy and omentectomy is controversial as studies have shown that there is no added benefit with the procedure [13, 14]. This is due to the fact that MOGCT are highly chemo-sensitive tumours.

EP was the most common chemotherapeutic regimen used upfront among our patients. We used to prescribe BEP initially after assessing the patient’s baseline pulmonary function status including diffusing capacity of the lungs for carbon monoxide (DLCO). However, when one patient had grade 5 pulmonary toxicity, we changed our departmental practice and started using the EP instead of BEP more frequently, wherever applicable. For FIGO stage I disease who required systemic therapy, EP was given for three cycles and it was given for four cycles for high-risk cases (FIGO stage II and above).

The projected 5-year PFS of the study population was 79.3% which is less than that of Agarwal *et al* [8] where it was reported to be 87%. This difference can be attributed to the fact that in their group dysgerminoma was the most common histology. However, the 5-year OS of our study is similar to that of Agarwal *et al* [8] and Newton *et al* [15].

Delayed normalisation of AFP is reported in the literature and it is usually associated with poor prognosis in the form of early recurrence [13]. Five patients had elevated AFP after treatment completion in our cohort and three had normalisation of AFP over time without any intervention. They did not have any recurrence so far till the date of last follow-up.

The most common toxicity in our study was febrile neutropenia which is similar to that of Talukdar *et al* [9]. One (1.6%) patient had grade 5 pulmonary toxicity who received BEP regime. Pulmonary toxicity of all grades is a serious complication of bleomycin and it is reported to be around 10% and mortality being reported around 2%–3% of all patients who have been treated with bleomycin [14]. Three (5%) patients developed features of metabolic syndrome. Metabolic syndrome has been reported in testicular germ cell tumours [15]. In men, platinum induced microvascular injury along with hypogonadism predisposes to the development of metabolic syndrome [3]. Proper detection and early intervention for the same is necessary as patients with MOGCT are expected to survive for a long time.

Being a retrospective study, the study has some limitations. However, it does have strong points. The study made an attempt to capture the treatment pattern of patients with MOGCT in a resource-limited setting like India. All patients were treated with standard systemic chemotherapy and we attempted to show treatment-related response rate and its adverse effect. A modified intent to treat survival analysis was made to avoid censoring.

## Conclusions

Immature teratoma was the most common tumour in our study population. With a combined modality of treatment, it is a highly curable malignancy with high PFS and OS rate. These tumours should be ideally evaluated by a trained gynaecological oncologist and further managed appropriately by specialised medical oncologists. Toxicity of the systemic therapy is manageable in most cases and should be carefully monitored. Our study reflects the real-world scenario of MOGCT from a limited resource low middle-income country like India.

## Conflicts of interest

None.

## Funding

None.

## Consent

Institutional Review Board waiver for consent was obtained (EC/WV/TMC/38/20).

## Figures and Tables

**Table 1.** Baseline characteristics and treatment details of the patient.

Characteristics	Value
Age	25 (11–58) years
Symptoms Abdominal pain Bleeding Abdominal distension Obstructive Incidental	52 (78.4%) 9 (13.8%) 12 (18.4%) 3 (4.6%) 3 (4.6%)
ECOG performance status 0 1 2 3 4	38 (58.4%) 15 ( 23%) 10 ( 15.3%) 1 (1.5%) 1 (1.5%)
Histology Dysgerminoma Immature teratoma grade 1 grade 2 grade 3 Yolk sac Mixed histology	13 (20%) 3 (4.6%) 6 (9.2%) 14 (21.5%) 22 (33.8%) 7 (10.7%)
FIGO stage IA IB IC II III IV	8 (12.3%) 2 (3%) 32 (49.2%) 2 (3%) 17 (26%) 4 (6.1%)
Tumour markers AFP (ng/mL) beta HCG m IU /mL LDH (U/L)	6553.9 +/− 1655 62.37 +/− 38.71 657.77 +/− 94.99
Surgery Upfront Post NACT	49 16
Type of surgery USO USO + omentectomy USO + omentectomy + nodal dissection BSO BSO + omentectomy Non-fertility preserving surgery	23 12 12 2 2 14
Type of systemic treatment Surveillance EP BEP	8 (14%) 37 (63%) 12 (23%)
Median cycle number	3 (3–4)

ECOG, Eastern Cooperative Oncology Group; NACT, Neoadjuvant chemotherapy; USO, Unilateral salpingo-oophorectomy; BSO, Bilateral salpingo-oophorectomy

**Table 2.** Comparison of characteristics between other studies and ours.

Characteristics	Agarwal *et al* [8]	Lakshmanan *et al* [11]	Topuz *et al* [1]	Newton *et al* [15]	Our study
Patient number	50	39	41	138	65
Median age	20.5	22	25	NA	25
Most common histology	Immature teratoma	Dysgerminoma	Dysgerminoma	Dysgerminoma	Immature teratoma
Most common stage	I	III	IA	IA	IC
DFS	87.5% at 5 years	Median not reached	Not mentioned	72%	79.3% at 5 years
